# Practical partnered research to improve weight loss among overweight/obese veterans: lessons from the trenches

**DOI:** 10.1186/s12874-017-0321-9

**Published:** 2017-03-29

**Authors:** Mona AuYoung, Laura J. Damschroder, Linda Kinsinger, Tannaz Moin, Caroline R. Richardson

**Affiliations:** 1 Ann Arbor VA Center for Clinical Management Research, VA HSR&D/CCMR (Mail Stop 152), P.O. Box 130170, Ann Arbor, MI 48113-0170 USA; 2 VA Diabetes QUERI, VA HSR&D/CCMR (Mail Stop 152), P.O. Box 130170, Ann Arbor, MI 48113-0170 USA; 3 National Center for Health Promotion and Disease Prevention, 3022 Croasdaile Dr #200, Durham, NC 27705 USA; 4 VA Greater Los Angeles Healthcare System, Los Angeles, CA USA; 5 David Geffen School of Medicine, University of California, Los Angeles, CA USA; 6 Greater Los Angeles CA Health Services Research and Development (HSR&D) Center for Healthcare Innovation, Implementation and Policy, 11301 Wilshire Blvd, Mail Code 111D, Los Angeles, CA 90073 USA; 7 Department of Family Medicine, University of Michigan, 1018 Fuller St, Ann Arbor, MI 48104-1213 USA

**Keywords:** Partnered research, Weight loss, Implementation, Pragmatic clinical trials, Veterans

## Abstract

**Background:**

Obesity and obesity-related conditions, such as type 2 diabetes, are a major issue for Veteran health. Veterans Health Administration (VA) researchers and health systems leaders have worked separately and together to provide more effective weight management programs for Veterans. Although randomized clinical trials are often considered the gold standard for establishing efficacy of interventions in controlled circumstances, pragmatic clinical trials (PCTs) provide agility for translation.

**Main text:**

VA researchers and health system leaders collaboratively designed a PCT to compare the Diabetes Prevention Program (VA-DPP) to usual care (MOVE!®) in promoting weight loss and glycemic control among overweight/obese Veterans with prediabetes. Together, they navigated the tensions that exist between quality improvement and research activities, facing challenges but reaping significant rewards. Early findings led to updated national guidance for delivering obesity treatment in VA.

**Short conclusion:**

Partnered research and the use of PCTs can be powerful strategies for accelerating evidence-based findings into practice. Collaborative partnerships between researchers and health systems leaders can help enhance and sustain translation in real-world settings.

## Background

It is essential that researchers work more closely with health system leaders such as program leaders, policy makers and other decision makers, to collaboratively design and conduct studies in a way that translates evidence-based programs more quickly and effectively into clinical practice [1–4]. However, differences in perspectives and priorities regarding the relative merits of alternative research questions and the timeline by which to answer these questions can introduce unique challenges and tensions. For example, researchers are rewarded for increasing generalizable knowledge in the scientific literature, which may take years to produce, while program leaders must focus on solving more specific problems and addressing more immediate organizational needs [5]. Research typically follows a slower timeline, whereas program changes may occur more rapidly and/or in real-time. This tension is exacerbated by research funding being more available for traditional clinical trials that focus on testing interventions in relatively controlled circumstances rather than assessing effectiveness and implementation approaches in less controlled real-world settings [6–9]. Frenk (1992) and others have described this tension as navigating trade-offs between scientific excellence (contributing to internal validity) versus being relevant, responsive, and timely (contributing to external validity) [4, 5, 10–13]. It can take an average of 17 years to translate clinical trial findings broadly into practice [14] and the challenges and delays are even greater for psychosocial treatments (e.g., behavioral interventions related to weight management) [15].

One of the main challenges for a successful partnership between researchers and health system leaders is finding a way to simultaneously meet competing needs of researchers and leaders. The nature of how the partnership was created or how it functions can impact the ability of the partners to navigate challenges. Partnerships with equal participation of both partner groups are better positioned to handle obstacles compared to partnerships where the organizational partner has a less equal role [2]. Researchers need time to develop research questions and study materials, obtain approval from ethics boards, hire staff, recruit participants and control the study environment in order to minimize bias. Health system leaders face enormous demands to get results implemented quickly to meet organizational demand. Frenk (1992) describes this as the difference between political time (perceived as warp speed) and scientific time (perceived as the pace of the proverbial tortoise) [5]. Moreover, decision makers often seek answers to more specific questions than those that traditional research offers; they are looking for a solution to a problem within their organization and not necessarily one that could potentially solve similar problems across multiple organizations [7]. However, research and decision makers can work to balance these perspectives by assessing multiple outcomes that are important to both partners and within the timeframe needed [8].

Researchers and funders have called for more pragmatic (or practical) clinical trials that generate knowledge that is essential to health system leaders who make real-world decisions based on e.g., cost, feasibility, fit, and likelihood of generating sufficient value for the investment [2, 3]. Although randomized controlled trials have long been considered the gold standard for evidence-based medicine, pragmatic trials may better answer these context-specific questions that are important in real-world implementation [16]. In order to facilitate pragmatic clinical trials, there must be existing infrastructure that enables interactions between researchers and decision makers and also helps to promote a solid working relationship before embarking on a research project together [5, 7]. Pragmatic trials focus on leveraging existing resources whenever possible to make implementation more feasible. These resources include the use of existing clinic staff (instead of separate or newly hired research staff), usual care comparison (instead of adding a program for a control group), and integrating research data into clinical practices (instead of having outside staff collect and manage data) [17]. Although pragmatic trials face a number of barriers, including insufficient funding (since they can be large studies with long follow-up periods) or sample sizes that are too small to detect treatment effects (because the unit of analysis is often at the organizational level e.g., practice-level), these may be outweighed by tremendous benefits [7].

This paper describes the evolution of a partnership between Veterans Health Administration (VHA) Diabetes Quality Enhancement Research Initiative (QUERI) center researchers and national health system leaders within the VHA National Center for Health Promotion and Disease Prevention (NCP), a center that has responsibility for developing policies to promote prevention in VHA, including weight management services. This collaborative partnership, which spans over 5 years and five research projects, has culminated most recently in a pragmatic clinical evaluation from which preliminary results were used to inform system-wide changes within the delivery of VA weight management services. Overall, this work provides a case illustration for how rapid, responsive, and relevant research findings [3] can be generated through a productive partnership between researchers and health system leaders. In addition to demonstrating the power of partnered research, this paper describes how to overcome challenges in conducting more pragmatic research within a clinical setting.

### A short history of weight management in the VA

As policy makers, NCP oversees health promotion within the VA, which spans guidance for clinical practices to providing programs and resources, including obesity prevention and weight management services. Over the past decade, NCP has faced a high priority need to address the growing obesity epidemic within VHA since 75% of Veterans using VA medical services were considered overweight or obese [18]. In response, NCP developed the evidence-based MOVE!® Weight Management Program for Veterans (hereafter referred to as “MOVE!”), which has been implemented at 155 VA medical centers and 872 community-based outpatient clinics across the nation [18]. Implementation of MOVE! varies widely across sites, although in general, program components include an initial assessment and weekly group sessions for 6 to 10 weeks [19]. The group sessions are intended to provide self-management support to help patients lose weight.

### An evolving research-organizational stakeholder partnership

In 2011, executive leaders in VA directed NCP to disseminate the Diabetes Prevention Program [20] in VA facilities within a very short timeframe. This request was motivated by the success of the Diabetes Prevention Program (DPP) which showed that compared to placebo, an intensive lifestyle change program was associated with a 58% reduction in type 2 diabetes incidence among individuals with pre-diabetes over 3 years of follow-up [20]. Long-term observational follow-up studies showed that these benefits were also sustained for up to 15 years, although results have been less dramatic in real-world settings where degree of implementation may vary [21–25].

NCP reached out to VA QUERI researchers to help conduct and evaluate a clinical demonstration of a group-based version of the DPP (VA-DPP) at three pilot sites in the VA. The VA QUERI researchers had experience and interest in developing and evaluating programs related to weight loss and chronic disease self-management. NCP leaders have collaborated with these researchers several times since the early days of the MOVE! program [26–29].

Because of this long-standing partnership and mutual trust built over time, the partnered team successfully negotiated challenging logistics to launch the VA-DPP demonstration only 10 months later. Since research partners often bring different perspectives in how to approach the project, the existing trusting relationship was a key factor in negotiating these differences to rapidly proceed with the VA-DPP trial [30]. (see Table 1) The challenges included funding, the short timeframe, and the need to navigate regulatory requirements governing clinical quality improvement (QI) initiative and research activities [31].

**Table 1:** Key decision points for partnered research design [30]

Design element	Operational considerations	Researcher considerations	Compromise
Study population			
Patient screening	No systematic pre-diabetes screening in place, just weight management referral system	Need to screen patients at risk for pre-diabetes	Use weight management referral system and add pre-diabetes screening
Study design			
Primary goal	Answer operational questions in real-time	Answer research questions over time	Answer both; program implemented on more rapid timeline and preliminary data used to make operational decisions
Group assignment	By site; pre-post	Randomization	Systematic assignment
Control group	Usual care	Control group	Usual care as control group
Resources	• Infrastructure • Separate funding • Leadership • Dissemination	• Research staff • Research funding • Program evaluation	Share resources
QI vs research			
Ethics review	IRB and informed consent not necessary for QI	IRB and informed consent required for research	IRB and informed consent for evaluation components only
Funding sources	Clinical funds for QI components	Research funds for evaluation components	Maintain separate funding sources for QI vs research; funding sources were pieced together over time
Outcome assessment			
Timeline	Need for rapid results	Need time for study design, proposal review, and data analysis	Provide preliminary results prior to final outcomes

### Navigating trade-offs

#### Study design

NCP leaders funded a clinical demonstration of the VA-DPP at three medical centers. This initiative was deemed a clinical QI project, which meant that implementation of the VA-DPP could proceed without the additional time and work needed to obtain institutional review board (IRB) approval; with pressure from high-level leaders to launch the project quickly, this was a key “win.” However, the researchers wanted to collect additional measures (e.g., motivation, goal setting) and link this information to administrative data (including clinical outcomes of change in weight and blood glucose) to better explain and potentially predict outcomes. To do this, research funding and IRB approval were needed for each study site without delaying the launch of the clinical program. Another crucial decision was the number of study sites: NCP leaders wanted to conduct the evaluation at three sites and identify three additional sites as controls. The research team was concerned about the ability to attribute any potential differences in outcomes to the program (DPP vs MOVE!) rather than significant differences between sites. On the other hand, having both the VA-DPP and MOVE! Conducted at the same site introduced other potential biases (e.g., spillover effect). The teams weighed the options and together, decided that the latter was a stronger design.

Both MOVE! And the VA-DPP were delivered in-person at three VA Medical Centers; the main differences were that the VA-DPP convened closed groups (participants progressed through the program in cohorts; no new patients could join after the program started), all sessions were led by a consistent facilitator, and VA-DPP was designed to deliver more sessions (16 over first 6 months vs 8–10 over first 6 months). Further details about both programs are published elsewhere [32, 33].

The VA-DPP was integrated into existing clinical processes to the extent possible. For example, the VA-DPP relied on existing MOVE! referral infrastructure and processes to recruit patients. Inclusion criteria also relied on weights and hemoglobin (HbA1c) test results that were documented as part of routine clinical care. However, the existing referral infrastructure was based on screening patients by body mass index, so each site had to add a procedure to also screen patients for pre-diabetes to determine study eligibility [32, 34–36]. This process was more complicated and time-consuming than expected [32, 34–36]. Each of the three sites had to design, often negotiate, and integrate a process to determine diabetes status (normal glycemic status, prediabetes, or diabetes). Compounding this challenge, the study sites experienced lower than expected numbers of referrals for weight management [37], on which recruiting for VA-DPP relied.

The three VA-DPP demonstration sites were selected to diversify the racial and ethnic mix of patients as well as geographic location. Exclusion criteria were minimal to allow for a more heterogeneous study population that reflected a typical real-world patient population. Within the setting and objectives of this evaluation, a randomized controlled clinical trial provides strong internal validity, avoiding potential bias that may occur with other assignment approaches. However, NCP leaders and local staff deemed randomization infeasible, as is often the case within naturalistic clinical practice settings. The teams collaboratively designed an approach to *systematically* assign patients to the VA-DPP vs. MOVE! (e.g., the first 20 referred eligible patients were assigned to VA-DPP and the second 20 patients assigned to MOVE!) [34]. Systematic assignment was a simple approach that clinical staff could use to divide eligible participants into two study arms. However, there were significant differences in race/ethnicity between the two assignments (p = 0.04): a higher proportion of Hispanic participants (8.8% vs 3.3%) and lower proportions of non-Hispanic black (36.3% vs 43.6%) and non-Hispanic white (39.8% vs 44.7%) participants were assigned MOVE! [35]. It is impossible to know whether randomization would have mitigated this imbalance. Because the VA-DPP demonstration was deemed a clinical QI initiative, written informed consent was not required for participation in the either VA-DPP or MOVE!. However, written, informed consent was required for participation in questionnaires and interviews conducted as a part of the multi-faceted evaluation.

#### Funding

Although clinical funding from NCP was used to conduct the implementation and day-to-day activities required for the actual demonstration of VA-DPP, research funding was needed to administer surveys to participants and to link survey data to clinical outcomes data (e.g., change in weight) for the purposes of evaluation. Each component of the overall project was individually deemed as being QI or research (the detailed list is published elsewhere) [33]; the research team had to obtain approval to use QI elements for the research analysis component of the project. The research evaluation was integrated with the QI project as a Hybrid Type III pragmatic effectiveness-implementation trial; i.e., the evaluation had a primary aim of studying a strategy for implementing VA-DPP and secondary aim of assessing clinical effectiveness [38]. Due to pressure to launch the VA-DPP project quickly, clinical activities were already underway when the research team submitted a proposal to conduct the evaluation under a “rapid response,” peer-reviewed, 1-year pilot grant mechanism in the VA [similar to a National Institutes of Health (NIH) R21 grant] to collect baseline survey data. Fortunately, the pilot grant was approved on the first submission, which enabled the research team to begin collecting baseline data just as enrollment into the demonstration started. The research team then submitted a large proposal for a multi-year grant mechanism (similar to an NIH R01 implementation grant) grant to fund the remaining 2 years of work required to collect follow-up data and conduct analyses. This too, fortunately, was reviewed and approved in its first submission.

#### Clinical vs research activities

As a clinical QI project, funded by clinical dollars, the VA-DPP demonstration project did not require approval by the IRB and as previously mentioned, written informed consent was not required to participate in either clinical program. Specifically, the delivery of the VA-DPP and MOVE! sessions, collection of weights and HbA1c were all part of the QI clinical process. However, *evaluation* of these data was considered research and required approvals by five IRBs at five different institutions: the three VA-DPP demonstration sites, the research coordinating site, and a research collaboration site. The IRBs were asked to approve use of VA-DPP QI data for the research evaluation. Written informed consent and signed HIPAA authorization were required to administer the questionnaires as well as to access and link clinical data (e.g., weights) with the questionnaire responses. The research team and the operational partners had to carefully navigate the complexities of determining whether activities were QI (related to the VA-DPP clinical demonstration) versus research (related to accompanying evaluation) throughout the project in order to ensure compliance to protocols (e.g., reporting adverse events, addressing missing data).

#### Timing

As previously mentioned, this pragmatic trial took place on an accelerated timeline. Midway through the trial, NCP was under pressure to brief senior leaders and congressional staff on progress and preliminary findings from the clinical demonstration even though the researchers were not yet comfortable releasing any findings so early in the process. The endeavor attracted the attention of the press, including Minnesota Public Radio (MPR) [39]. Rapid dissemination of preliminary research results can be a double-edged sword for researchers. While the immediate attention on research findings by high-level leaders and the press helped to rapidly and broadly publicize the initiative, there is the risk that final outcomes may differ from preliminary findings. In this case, preliminary findings indicated that participants in the VA-DPP study arm experienced greater weight loss than those in the control arm (at 6 months). However, final outcomes indicated that by 12 months, though trends were in the same direction, program differences were no longer significant.

### Translating findings into clinical practice

The VA-DPP trial findings were rapidly translated to update guidance to the field to bring the pre-existing MOVE! program more closely in line with features of VA-DPP. These included recommendations for a single, consistent session leader rather than rotating topic experts, closing groups to new patients after a cohort started rather than allowing patients to enter the program midway through the series, and extending the program to at least 16 sessions from 12. The MOVE! program guidelines were updated prior to completion of the evaluation and before publication of outcomes. The pragmatic nature of the VA-DPP trial provided context-sensitive information to help inform rapid translation of results into clinical settings. This approach differs significantly from the traditional research process with significant lag times between trial completion and publication of findings and then practitioners attempting to translate findings into their own settings. However, this experience validates the “integrated, contextual, multilevel research-practice integration systems approach” described by Glasgow and Chambers (2012) [3]. (see Table 2) The evolution of the MOVE! program over the last decade is a testament to the learning approach taken by NCP in its pursuit of continuing to improve weight management programming for Veterans [40].

**Table 2:** Examining rigor and relevance of the VA-DPP implementation study [3]

	Characteristic	VA-DPP
Systems perspective		
1	Context is critical	VA-DPP intervention is clearly focused on preventing diabetes among Veterans with pre-diabetes within an integrated health system
2	Multilevel complexity	Diabetes prevention involves multi-component, comprehensive lifestyle interventions; the VA-DPP coaches deliver personalized treatment
3	Focus on systems characteristics	VA health system leaders supported the program and efforts were made to gain buy-in from VA site leaders and staff; implementation and delivery were tailored to sites
Robust, practical goals		
4	Representativeness and reach	Minimal eligibility criteria to reach wider population of Veterans in need
5	Generalizability	Designed to assess generalizability of the DPP’s results for the largely male Veteran population
6	Pragmatic and practical	Showed VA leaders the feasibility of implementing this program in the VA; it also showed the potential effectiveness of VA-DPP for Veterans
7	Scalability and sustainability	The VA-DPP was piloted at three sites in order to determine feasibility across all VA sites nationally designed to utilize existing clinical processes when possible in order to promote sustainability, although existing clinical processes limited recruitment
Research methods to enhance relevance		
8	Rigorous	The VA-DPP maintained scientific rigor by using systematic assignment to intervention arms, but also focused on replicability by integrating the study into existing clinical processes as much as possible
9	Rapid	VA health system leaders decided to make systemic changes based on early findings (at 6 months) indicating greater weight loss with the VA-DPP
10	Adaptive	This research partnership was based on previous collaborations on similar weight loss interventions for Veterans
11	Integration of methods; triangulation	Quantitative data (patient weights, HbA1cs, questionnaires, fidelity checklists) and qualitative data (patient and staff interviews) were integrated to assess VA-DPP implementation and effectiveness
12	Relevance	The VA-DPP was a high priority for senior leaders
Flexibility		
13	Multiplicity	The partnership between researchers and health system leaders brought different viewpoints with how to implement the VA-DPP
14	Respect for diverse approaches; humility	The researchers and health system leaders collaboratively decided on the best approach for conducting the VA-DPP evaluation

### Benefits and challenges of partnered research for the health system leader

Having a strong relationship with an experienced and trusted group of researchers provides clear benefits for health system leaders, like those within NCP, who are responsible for developing and implementing policies and programs within a large national integrated healthcare organization. Program offices often do not have the staff, funding, or expertise needed to conduct complex evaluations on their own programs. Researchers are better prepared to take on the work of obtaining IRB approvals, hiring research assistants, mailing out surveys, collecting and analyzing data, and other research activities. Adding the research components complicated the study because of the additional data collection and evaluation processes leading to additional time and effort needed to obtain IRB approvals at five different sites and annual renewals for each. The researchers also needed to obtain funding from multiple sources (all with peer review) for the research components; this work had to be done in a way that did not interfere with quickly launching the demonstration project; in fact, the project was going to launch with or without the research funding. The research team was focused on minimizing bias as much as possible during the study. Additionally, the relative independence of the research group conducting the evaluation helps provide credibility for the results of the work. However, external pressure for findings resulted in system-level changes in guidance based on preliminary findings; much earlier than the research team would otherwise have recommended.

### Benefits and challenges of partnered research for the researcher

There are clear advantages to having a health systems leader as a research partner. NCP provided key support for the VA-DPP demonstration project, including technical expertise, insight into their priorities, and funding. They and the local VA sites also offered use of existing system infrastructure within which to conduct the VA-DPP evaluations for program referrals and clinical outcomes data. Although the research timeline was accelerated for the VA-DPP clinical demonstration, researchers maximized rigor by making careful trade-offs between strengthening internal versus external validity to inform study design and data collection. Having an engaged health system partner established high-level and local leadership support for the evaluation, which helped to reduce barriers that potentially could have slowed or delayed the project. Challenges included the need to carefully differentiate between research and clinical quality improvement activities to determine the appropriate actions, including whether IRB review and approval of amendments was needed. Although NCP’s contribution of clinical funding shows deep support and engagement, it can also give the impression of undue influence on the research questions, design and outcomes. Regular communications and transparency were essential to ensuring that all stakeholders are equal partners while maintaining independence to ensure scientific rigor. With NCP as an engaged research partner, there was the clear benefit of a built-in dissemination channel across VA sites nationally, which meant that research findings would be translated quickly with tangible results.

## Conclusions/Recommendations for productive partnerships in research

Partnered research, especially within the context of pragmatic clinical trials, can help to accelerate translation of evidence-based interventions into clinical practice. In our experience, both partners had a shared goal for research: learning how to evolve weight loss treatment for Veterans to continue to improve outcomes. The longstanding relationship between health system leaders and researchers was built on mutual trust and allowed each to adjust to conducting quality research yielding valuable information on a rapid timeline. The shared history of partnership contributed to establishing shared goals and clear lines of communication. Conducting partnered research can be a challenging process but can result in significant rewards. As research becomes more collaborative and funders continue to focus on applying and integrating findings into practice, this collaborative model of research may become more the norm, especially as the movement toward creating learning health systems grows.

## Abbreviations

DPP: Diabetes Prevention Program
IRB: Institutional review board
NCP: VHA National Center for Health Promotion and Disease Prevention
NIH: National Institutes of Health
QI: Quality improvement
VA: Veterans Affairs
VA-DPP: Veterans Affairs Diabetes Prevention Program
VHA: Veterans Health Administration

## References

[1] Kilbourne AM, Atkins D (2014). Partner or perish: VA health services and the emerging bi-directional paradigm. J Gen Intern Med.

[2] Sibbald SL, Tetroe J, Graham ID (2014). Research funder required research partnerships: a qualitative inquiry. Implementation science : IS.

[3] Glasgow RE, Chambers D (2012). Developing robust, sustainable, implementation systems using rigorous, rapid and relevant science. Clin Transl Sci.

[4] Riley WT, Glasgow RE, Etheredge L, Abernethy AP (2013). Rapid, responsive, relevant (R3) research: a call for a rapid learning health research enterprise. Clin Transl Med.

[5] Frenk J (1992). Balancing relevance and excellence: organizational responses to link research with decision making. Soc Sci Med.

[6] Ackermann RT, Duru OK, Albu JB, Schmittdiel JA, Soumerai SB, Wharam JF, Ali MK, Mangione CM, Gregg EW, Group N-DS (2015). Evaluating diabetes health policies using natural experiments: the natural experiments for translation in diabetes study. Am J Prev Med.

[7] Tunis SR, Stryer DB, Clancy CM (2003). Practical clinical trials: increasing the value of clinical research for decision making in clinical and health policy. Jama.

[8] Glasgow RE, Green LW, Klesges LM, Abrams DB, Fisher EB, Goldstein MG, Hayman LL, Ockene JK, Orleans CT (2006). External validity: we need to do more. Ann Behav Med.

[9] Glasgow RE, Vinson C, Chambers D, Khoury MJ, Kaplan RM, Hunter C (2012). National Institutes of Health approaches to dissemination and implementation science: current and future directions. Am J Public Health.

[10] Green LW (2008). Making research relevant: if it is an evidence-based practice, where's the practice-based evidence?. Fam Pract.

[11] Green LW, Glasgow RE (2006). Evaluating the relevance, generalization, and applicability of research: issues in external validation and translation methodology. Eval Health Prof.

[12] Godwin M, Ruhland L, Casson I, MacDonald S, Delva D, Birtwhistle R, Lam M, Seguin R (2003). Pragmatic controlled clinical trials in primary care: the struggle between external and internal validity. BMC Med Res Methodol.

[13] Fransen GA, van Marrewijk CJ, Mujakovic S, Muris JW, Laheij RJ, Numans ME, de Wit NJ, Samsom M, Jansen JB, Knottnerus JA (2007). Pragmatic trials in primary care. Methodological challenges and solutions demonstrated by the DIAMOND-study. BMC Med Res Methodol.

[14] Morris ZS, Wooding S, Grant J (2011). The answer is 17 years, what is the question: understanding time lags in translational research. J R Soc Med.

[15] IOM (Institute of Medicine). Psychosocial interventions for mental and substance use disorders: A framework for establishing evidence-based standards. Washington, DC: The National Academies Press; 2015. 26203478

[16] Goldenberg MJ (2009). Iconoclast or creed? Objectivism, pragmatism, and the hierarchy of evidence. Perspect Biol Med.

[17] Thorpe KE, Zwarenstein M, Oxman AD, Treweek S, Furberg CD, Altman DG, Tunis S, Bergel E, Harvey I, Magid DJ (2009). A pragmatic-explanatory continuum indicator summary (PRECIS): a tool to help trial designers. J Clin Epidemiol.

[18] Kinsinger LS, Jones KR, Kahwati L, Harvey R, Burdick M, Zele V, Yevich SJ (2009). Design and dissemination of the MOVE! Weight-management program for veterans. Prev Chronic Dis.

[19] Kahwati LC, Lewis MA, Kane H, Williams PA, Nerz P, Jones KR, Lance TX, Vaisey S, Kinsinger LS (2011). Best practices in the Veterans Health Administration's MOVE! Weight management program. Am J Prev Med.

[20] Knowler WC, Barrett-Connor E, Fowler SE, Hamman RF, Lachin JM, Walker EA, Nathan DM, Diabetes Prevention Program Research G (2002). Reduction in the incidence of type 2 diabetes with lifestyle intervention or metformin. N Engl J Med.

[21] Diabetes Prevention Program Research G (2015). Long-term effects of lifestyle intervention or metformin on diabetes development and microvascular complications over 15-year follow-up: the Diabetes Prevention Program Outcomes Study. Lancet Diabetes Endocrinol.

[22] Knowler WC, Fowler SE, Hamman RF, Christophi CA, Hoffman HJ, Brenneman AT, Brown-Friday JO, Goldberg R, Venditti E, Diabetes Prevention Program Research G (2009). 10-year follow-up of diabetes incidence and weight loss in the Diabetes Prevention Program Outcomes Study. Lancet.

[23] Aziz Z, Absetz P, Oldroyd J, Pronk NP, Oldenburg B (2015). A systematic review of real-world diabetes prevention programs: learnings from the last 15 years. Implementation science : IS.

[24] Ali MK, Echouffo-Tcheugui J, Williamson DF (2012). How effective were lifestyle interventions in real-world settings that were modeled on the Diabetes Prevention Program?. Health Aff (Millwood).

[25] Gregg EW, Ali MK, Albright A (2014). Comment on Kahn and Davidson. The reality of type 2 diabetes prevention. Diabetes Care 2014;37:943-949. Diabetes Care.

[26] Damschroder LJ, Goodrich DE, Robinson CH, Fletcher CE, Lowery JC (2011). A systematic exploration of differences in contextual factors related to implementing the MOVE! weight management program in VA: a mixed methods study. BMC Health Serv Res.

[27] Littman AJ, Damschroder LJ, Verchinina L, Lai Z, Kim HM, Hoerster KD, Klingaman EA, Goldberg RW, Owen RR, Goodrich DE (2015). National evaluation of obesity screening and treatment among veterans with and without mental health disorders. Gen Hosp Psychiatry.

[28] Damschroder LJ, Lowery JC (2013). Evaluation of a large-scale weight management program using the consolidated framework for implementation research (CFIR). Implementation science : IS.

[29] Damschroder LJ, Reardon CM, Sperber N, Robinson CH, Fickel JJ, Oddone EZ. Implementation evaluation of the telephone lifestyle coaching (TLC) program: organizational factors associated with successful implementation. Transl Behav Med*.* In press. 10.1007/s13142-016-0424-6 PMC5526796 27688249

[30] Bauer MS, Miller C, Kim B, Lew R, Weaver K, Coldwell C, Henderson K, Holmes S, Seibert MN, Stolzmann K (2016). Partnering with health system operations leadership to develop a controlled implementation trial. Implementation science : IS.

[31] Petzel RA (2011). VHA Operations Activities That May Constitute Research.

[32] Moin T, Damschroder LJ, AuYoung M, Maciejewski ML, Datta SK, Weinreb JE, Steinle N, Billington C, Hughes M, Makki F, et al. Diabetes prevention program translation in the veterans health administration. American Journal of Preventive Medicine. In press. 10.1016/j.amepre.2016.11.009 PMC6699500 28094135

[33] Damschroder LJ, Moin T, Datta SK, Reardon CM, Steinle N, Weinreb J, Billington CJ, Maciejewski ML, Yancy WS, Hughes M (2015). Implementation and evaluation of the VA DPP clinical demonstration: protocol for a multi-site non-randomized hybrid effectiveness-implementation type III trial. Implementation science : IS.

[34] Moin T., Damschroder LJ, Youles B., Makki F, Billington C, Yancy W, Maciejewski ML, Kinsinger LS, Weinreb J, Steinle NE, et al. Implementation of a prediabetes identification algorithm for overweight and obese Veterans. J Rehabil Res Dev*.* In press. 10.1682/JRRD.2015.06.0104 28273326

[35] Moin T, Richardson CR, Damschroder LJ. Implementing diabetes prevention in the VA: results from a clinical demonstration project. San Antonio: Paper presented at Society of Behavioral Medicine Annual Meeting and Scientific Sessions; 2015.

[36] Damschroder LJ, Reardon CM, AuYoung M, Moin T, Datta SK, Sparks JB, Maciejewski ML, Steinle N, Weinreb JE, Hughes M. et al. Evaluation of the VA Diabetes Prevention Program (VA-DPP) Clinical Demonstration in the Veterans Health Administration (VHA): Implementation Findings. Under review. 10.1186/s13012-017-0619-3 PMC5530572 28747191

[37] Kahwati LC, Lance TX, Jones KR, Kinsinger LS (2011). RE-AIM evaluation of the Veterans Health Administration's MOVE! Weight Management Program. Transl Behav Med.

[38] Curran GM, Bauer M, Mittman B, Pyne JM, Stetler C (2012). Effectiveness-implementation hybrid designs: combining elements of clinical effectiveness and implementation research to enhance public health impact. Med Care.

[39] Mador J (2012). New VA program targets diabetes in vets.

[40] Kinsinger LS (2015). Disease prevention in the Veterans Health Administration. N C Med J.

